# Autism-like behaviors regulated by the serotonin receptor 5-HT2B in the dorsal fan-shaped body neurons of *Drosophila melanogaster*

**DOI:** 10.1186/s40001-022-00838-1

**Published:** 2022-10-17

**Authors:** Haowei Cao, Junbo Tang, Qisha Liu, Juan Huang, Rui Xu

**Affiliations:** 1 State Key Laboratory of Reproductive Medicine, Center of Global Health, Nanjing Medical University, Nanjing, 211166 China; 2 Key Laboratory of Pathogen Biology of Jiangsu Province, Department of Pathogen Biology, Nanjing Medical University, Nanjing, 211166 China; 3 Jiangsu Key Laboratory of Brain Disease and Bioinformation, Research Center for Biochemistry and Molecular Biology, Xuzhou Medical University, Xuzhou, 221004 China; 4 Capital Medical University School of Basic Medical Sciences, Beijing, 100069 China; 5 College of Food Science and Nutritional Engineering, China Agricultural University, Beijing, 100193 China

**Keywords:** *Drosophila melanogaster*, Autism, Social space, Repetitive behavior, Serotonin, *5-HT2B*

## Abstract

**Background:**

Autism spectrum disorder (ASD) is a neurodevelopmental disorder characterized by impairments in social interaction and repetitive stereotyped behaviors. Previous studies have reported an association of serotonin or 5-hydroxytryptamine (5-HT) with ASD, but the specific receptors and neurons by which serotonin modulates autistic behaviors have not been fully elucidated.

**Methods:**

RNAi-mediated knockdown was done to destroy the function of tryptophan hydroxylase (Trh) and all the five serotonin receptors. Given that ubiquitous knockdown of *5-HT2B* showed significant defects in social behaviors, we applied the CRISPR/Cas9 system to knock out the *5-HT2B* receptor gene. Social space assays and grooming assays were the major methods used to understand the role of serotonin and related specific receptors in autism-like behaviors of *Drosophila melanogaster.*

**Results:**

A close relationship was identified between serotonin and autism-like behaviors reflected by increased social space distance and high-frequency repetitive behavior in *Drosophila.* We further utilized the binary expression system to knock down all the five 5-HT receptors, and observed the 5-HT2B receptor as the main receptor responsible for the normal social space and repetitive behavior in *Drosophila* for the specific serotonin receptors underlying the regulation of these two behaviors. Our data also showed that neurons in the dorsal fan-shaped body (dFB), which expressed 5-HT2B*,* were functionally essential for the social behaviors of *Drosophila*.

**Conclusions:**

Collectively, our data suggest that serotonin levels and the 5-HT2B receptor are closely related to the social interaction and repetitive behavior of *Drosophila*. Of all the 5 serotonin receptors, 5-HT2B receptor in dFB neurons is mainly responsible for serotonin-mediated regulation of autism-like behaviors.

**Supplementary Information:**

The online version contains supplementary material available at 10.1186/s40001-022-00838-1.

## Introduction

In humans, autism spectrum disorder (ASD) is a neurodevelopmental disorder characterized by a suite of key behavioral anomalies, consisting of repetitive behavior [1], abnormal verbal communication [2], hyperactivity [3], sensory abnormalities [4], and impaired social interactions [5]. The whole blood serotonin levels (WB 5-HT) were elevated in 28.3% of whole blood (WB) and 22.5% of platelet-rich plasma (PRP) samples of autistic individuals [6], and serotonin was found to play a crucial role in simulating cell proliferation in brain development in early childhood [6]. Although elevation of serotonin is reported in the blood, its level in the brain is relatively lower in autistic children [7–9]. It is noteworthy that maternal virus exposure or immune activation could also result in low-level brain 5-HT or abnormal 5-HT neurons [10, 11]. Despite its vital role in the brain, relatively little is known about the mechanisms by which 5-HT regulates autistic behaviors, especially the role of 5-HT receptors.

Fruit flies are social animals [12]. They communicate with others to compete for various resources, such as food [13] or reproductive partners [14], and synchronize their daily activities to one another by chemicals [15], as well as auditory [16] and tactile cues [17]. Social experiences could affect the behavior patterns of individuals and their neighbors, such as social learning and memory [18], social synchronizing (activities and rests) [19], aggression and mating [20]. Repetitive and stereotyped behaviors are common in people with autism, and patients show repeated hand clapping and finger shaking in front of the eyes [21]. Thus, repetitive and stereotyped behavioral characteristics are also considered a diagnostic criterion for autism [22]. The grooming behavior of *Drosophila* is an orderly repetitive motion that is similar to the repetitive and stereotyped behavior in autism. Therefore, we used a grooming assay to assess the repetitive behavior of *Drosophila* in this study. There are only approximately ninety 5-HT neurons in the *Drosophila* central brain [23], which makes *Drosophila* a tractable model to investigate how 5-HT modulates social behaviors.

Here, to examine whether *Drosophila* 5-HT neurons contribute to their social behaviors, we employed a social space assay to detect social interaction and a grooming assay to reflect the repetitive behavior of *Drosophila*. The receptor genes involved in autism-like behaviors were identified by combination with RNAi-mediated knockdown. Our study showed that serotonin levels affect the social interaction and repetitive behavior of *Drosophila* through the 5-HT2B receptor. We further reported that serotonin could regulate the two social behaviors through a small subset of 5-HT2B-expressing neurons in the dFB of *Drosophila* by specific knockdown 5-HT2B. Together, our work reveals that serotonin regulates the social interaction and repetitive behavior of *Drosophila* through the 5-HT2B receptor in dFB neurons.

## Materials and methods

### Fly stocks and rearing conditions

The fly lines used as controls were wild-type *Canton-S* and *w*^*1118*^, depending on the different genetic backgrounds of the test groups. All fly stocks were reared with normal standard yeast-cornmeal-agar medium in incubators at 25 °C and 60% humidity with a 12 h/12 h light/dark cycle. *Trh*^*01*^, *5-HT2B-Gal4*, *23E10-Gal4 *(hereafter *dFB-Gal4*, as it specifically expresses Gal4 in dorsal fan-shaped bodies [24]), *per-Gal4*, *or83b-Gal4*, and *Pdf-Gal4* lines were gifts from the Yi Rao Laboratory (Peking University, Beijing, China). Other *Gal4* and transgenic RNAi lines were obtained from Tsinghua Fly Center (THFC, Beijing, China), Bloomington *Drosophila* Stock Center (BDSC, Indiana University, USA) and Vienna *Drosophila* Resource Center (VDRC, Vienna, Austria), including *Tub-Gal4/TM6B* (THFC#TB00129), *Ubi-Gal4* (THFC#TB00152), *23E10-Gal4*(BDSC#49032), *or83b-Gal4(*BDSC#26818), *UAS-Trh-RNAi/TM3* (THFC#THU2052 or BDSC#25842), *UAS-5-HT1A-RNAi* (THFC#THU1216 or BDSC#33885), *UAS-5-HT1B-RNAi* (THFC#THU0772 or BDSC#33418), *UAS-5-HT2A-RNAi* (THFC#TH01471. N), *UAS-5-HT2B-RNAi* (VDRC#102356), *UAS-5-HT7-RNAi* (THFC#THU0916 or BDSC#32471).

### Construction of knockout lines of *Drosophila*

The fly line used for generating *5-HT2B* knockout transgene flies was *w*^*1118*^; *{nos-Cas9} attP40*/*CyO*, which was generated based on the *{nos-Cas9} attP40* line generously provided by Dr. Jianquan Ni (Tsinghua University, Beijing, China), whose X chromosomes were replaced by those of *w*^*1118*^. Cas9-mediated genome editing was performed as previously described [25]. Briefly, the gRNAs were designed with the help of the CRISPR Optimal Target Finder website (http://targetfinder.flycrispr.neuro.brown.edu/). The sgRNAs with no off-targets were chosen and subcloned into a pU6b-sgRNA-short vector (also kindly supplied by Dr. Ni) [25]. Two sgRNA plasmids targeting up- and down-stream sequences of the 5-HT2B start codon were injected into *{nos-Cas9} attP40* embryos. The knockout lines were screened by PCR analysis and subsequent DNA sequencing. Sequences of sgRNAs and screening primers used in line construction are recorded in Additional file 4: Table S1. The knockout lines were then backcrossed into a *w*^*1118*^ background for at least five generations.

### Pharmacological treatment of flies with 5-hydroxytryptophan (5-HTP)

5-HTP (Cat# H9772, Sigma) dissolved in ddH_2_O was mixed with freshly cooked and cooled standard fly food to make 2 mg/ml 5-HTP-containing food. For behavioral analysis, wild-type or *Trh*^*01*^ flies were maintained on normal food before the adult stage. Upon eclosing, adult flies were anesthetized on ice, separated according to their sex, and then transferred to 5-HTP food or standard food for 3–5 days. For immunostaining analysis, flies were dissected after 3 days of feeding with 5-HTP-containing food.

### Quantitative RT-PCR

Total RNA was extracted from 10 whole fly bodies using TRIzol reagent (Cat# 15596026, Invitrogen, Carlsbad, USA) according to the manufacturer’s instructions. Reverse transcription was performed using the HiScript III 1st Strand cDNA Synthesis Kit (+ gDNA wiper) (Cat# R312-01, Vazyme, Nanjing, China) following the manufacturer’s instructions. Real-time PCR was carried out on a real-time thermal cycler (QuantStudio 5, Thermo Fisher, MA, USA) using ChamQ SYBR qPCR Master Mix (Low ROX Premixed) (Cat# Q331-02, Vazyme), with three technical replicates for each sample. The PCR mix and qPCR program were prepared according to the manufacturer’s instructions. All the reactions were repeated at least three times, and analysis of the relative expression was performed by the ΔΔCT method. The data were validated by using the *RpL49* gene as an internal control. Primer sequences are listed in Additional file 4: Table S1.

### Serotonin detection

Approximately 50 whole bodies from 5- to 7-day-old flies were pooled and placed in 0.5 mL phosphate buffered saline (PBS) buffer solution, homogenized on ice for 5–10 min, and then centrifuged at 3000 rpm for 20 min to remove tissue debris. A serotonin assay kit (Cat# H104, Jiancheng, Nanjing, China) was used to perform ELISA to measure the 5-HT concentration of the supernatants. The final amount of 5-HT was normalized to the total weight of flies for each sample.

### Social space assay

A social space assay was conducted for social interaction analysis of grouped flies by using a previous protocol developed by Simon and coworkers [26]. Briefly, one day prior to the experiment, flies (30 < *n* ≤ 40) were collected and separated by sex under cold anesthesia. Before the experiment, the flies were transferred into new food vials and placed in a dedicated behavior room (50% humidity, 25 °C) to acclimate to the environment for 2 h. All experiments were performed in the largest triangular vertical chambers (inner dimensions: 16.5 cm by 16.5 cm by 14.5 cm). When flies settled in the chamber after approximately 30 min of exploration, digital images of the chamber were taken with a camera. Then, Fiji (ImageJ) software (NIH) was used to calculate the distance from the fly to its nearest neighboring fly, and GraphPad Prism 7.00 was used to analyze the data. For groups of flies with no climbing defects, we used vertically oriented chambers to perform social space analysis. However, for flies with climbing impairments, a potential remedy would be to use horizontal chambers or to allow more time for the flies to settle before taking a picture.

### Negative geotaxis assay

The locomotion of flies was tested by the negative geotaxis assay as previously described [27] with slight modifications. Briefly, 1 day before the experiment, adult male flies eclosed for 5–7 days were separated and placed in vials at a density of 25–30 flies per vial. During the experiment, the flies were transferred to glass tubes (inner diameter: 2 cm, height: 20 cm) without anesthesia. Then, the tubes were tapped by using a similar force to collect the flies to the bottom, and flies were then given 10 s to climb the wall. Digital images were taken, and the percentage of flies that crossed the 8 cm line on the wall within 10 s was calculated. This assay was repeated for the same group 3 times with a 1-min rest period for the tested flies between 2 trials. Similar procedures were repeated three times in total. The experiments were performed between 3 and 6 pm to minimize the potential effects of circadian oscillation.

### Repetitive behavior

Repetitive behavior was determined by the grooming assay, as previously described [28]. Generally, 5–7 days after eclosion, male flies were separated before the day of the experiment, and then a single fly was transferred into an observation chamber and placed in the behavior room for 2 h. Then, a 5-min video was recorded after flies acclimated in the chamber for 1 min. Data were collected for the number of individual grooming episodes. For some cases, the time of the fly spent grooming and the duration of individual grooming bouts were also collected. When a fly stopped grooming and kept motionless for 2 s or stopped grooming and walked at least 4 steps, grooming bouts were regarded as ending. In this experiment, we performed grooming experiments between 3 and 6 pm and manually analyzed the data during the 5-min observation period using video software.

### Immunohistochemistry and confocal imaging

For all immunostainings, adult female flies 5–7 days after eclosion fed with 5-HTP or normal food were anesthetized, and their brains were dissected in ice-cold PBS and fixed in 4% paraformaldehyde in PBS for 30 min at room temperature, followed by 4 rinses in PAT3 (0.5% Triton X-100, 0.5% bovine serum albumin in PBS) for 10 min at room temperature. Samples were transferred to 5% normal donkey serum (NDS) or normal goat serum (NGS) in PAT3 for 1 h of blocking at room temperature and incubated with primary antibodies (diluted in 5% NDS or NGS) at room temperature for 4 h and then at 4 °C overnight. After washing samples 4 times for 10 min with PAT3 at room temperature and incubating samples with secondary antibodies in 5% NDS or NGS at room temperature for 4 h and 4 °C overnight, we mounted the samples with FocusClear^™^ (Cat# FC-10100, CelExplorer Labs, Taiwan, China) and imaged them on a Zeiss LSM800 confocal microscope. The following antibodies were used: rabbit anti-5-HT (1:1000; Cat# 20080, RRID: AB_572263, ImmunoStar, Hudson, WI, USA) and mouse anti-nc82 (1:20; Cat# 2314866, RRID: AB_2314866, DSHB, USA). Secondary antibodies were diluted at 1:500 and were as follows: goat anti-rabbit Alexa Fluor 488 (Cat# A11008, RRID: AB_143165, Thermo Fisher Scientific, Foster City, CA, USA) and donkey anti-mouse Alexa Fluor 568 (Cat# A10037, RRID: AB_2534013, Thermo Fisher Scientific).

### Statistical analyses

GraphPad Prism 7.00 was applied for statistical significance analysis. The Kolmogorov–Smirnov test was used to analyze social space behavior, and a 2-tailed Student’s *t*-test for the data of 2 columns. The sample sizes are indicated in the figures. *P* values are denoted by *P < 0.05, **P < 0.01, ***P < 0.001, ****P < 0.0001 and NS (*P* ≥ 0.05). Exact *P* values are specified in the legends.

## Results

### Serotonin regulates social interaction in *Drosophila*

**Fig. 1:**
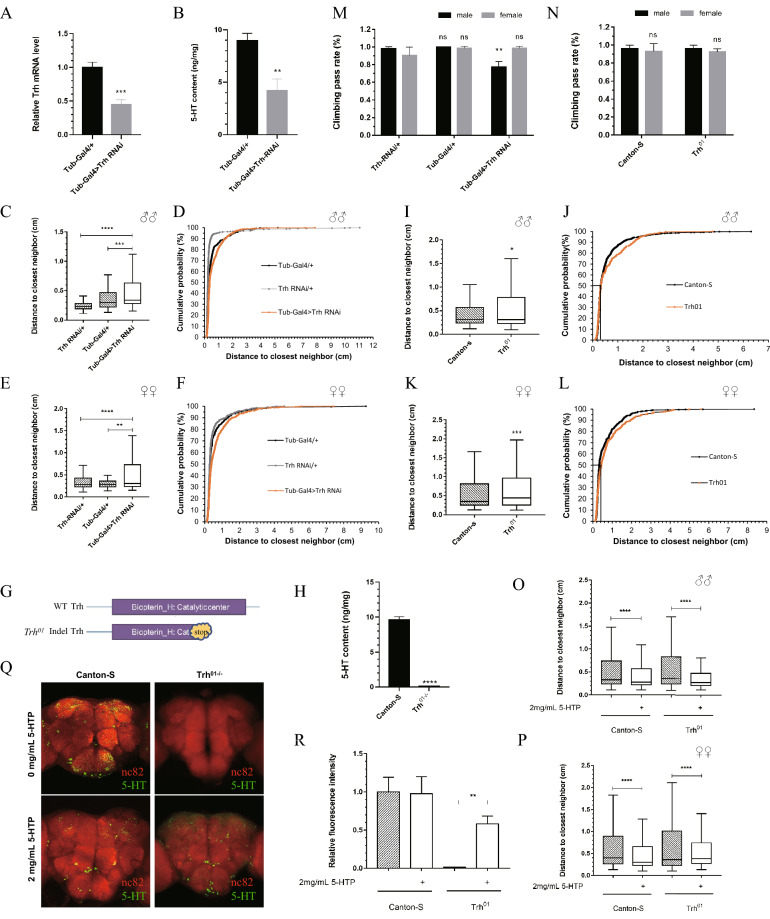
Serotonin regulates the social interaction of *Drosophila*. **A** qRT-PCR analysis of *Trh* mRNA expression in *Trh* knockdown lines. **B** Concentration of 5-HT in whole bodies of *Trh* knockdown and control flies. **C**, **E** Social spacing of males (**C)** and females (**E)** overexpressing *Trh-RNAi* with a *Tub-Gal4* driver. **D**, **F** Cumulative probability distributions of the closest neighbor distance in males (**D**) and females (**F**) overexpressing *Trh-RNAi* with a *Tub-Gal4* driver. **G** Schematic illustration of the WT *Trh* genome and the *Trh*^*01*^ indel mutant*,* whose catalytic center is deleted. **H** Concentration of 5-HT in whole bodies of *Canton-S* and *Trh*^*01*^ mutant flies. **I**, **K** Representative data show the distance to the closest neighbor of *Canton-S* and *Trh*^*01*^ mutant male (**I)** or female flies (**K**). **J**, **L** Cumulative probability distributions of the closest neighbor distance in *Canton-S* and *Trh*^*01*^ mutant males (**J**) and females (**L**). The number “50” on Y-axis and its corresponding number of *Thr*^*01*^ on X-axis are marked. **M**, **N** Negative geotaxis assay of *Trh* knockdown (**M)** and *Trh*^*01*^ (**M)** mutant flies. **O**, **P** *Canton-S* and *Trh*^*01*^ mutant individual flies displayed a decreased distance to the closest neighbor after feeding 2 mg/mL 5-HTP for 3 days. **Q** Brains of *Canton-S* and *Trh*^*01*^ mutant flies with or without 5-HTP feeding for 3 days immunostained with an anti-serotonin antibody (green) and the neuropil marker NC82 antibody (red). **R** The statistical analysis (mean ± SEM, at least *n* = 4 brains for each group) of the fluorescence intensity of the brains in **Q** measured by Fiji (ImageJ) software. Error bars are shown as the mean ± SEM (**A**, **B**, **H**, **M**, **N** and **R)**. Other data (**C**, **E**, **I**, **K**, **O** and **P)** are represented in a box and whisker plot of the distance to the closest neighbor in the chamber, with the box representing the 1st quartile (25th percent) and the 3rd quartile (75th percent), the line in the box representing the median, and Tukey’s whiskers excluding the outliers. These data were obtained from at least three independent repeats of 35 – 40 flies per assay. *p < 0.05, **p < 0.01, ***p < 0.001, ****p < 0.0001

The generation of serotonin requires a 2-step synthesis: the conversion of tryptophan to 5-HTP by TRH, followed by the aromatic amino acid decarboxylase-catalyzed conversion of 5-HTP to 5-HT [29]. To determine whether serotonin influences social interaction in *Drosophila*, we deleted serotonin by RNAi or using mutant lines. For *Trh* knockdown, we crossed virgin flies carrying *Tub-Gal4* drivers to male flies carrying *UAS-Trh-RNAi* transgenes for ubiquitous knockdown of *Trh* and verified the downregulation of *Trh* expression by qRT-PCR. The qPCR analysis showed that the mRNA expression of *Trh* was reduced by 55% in the RNAi lines (Fig. 1A). Then, we detected 5-HT levels by using the serotonin assay kit and found that *Tub-Gal4* > *UAS-Trh-RNAi* had significantly lower 5-HT levels (Fig. 1B). Impairment in social interaction is a major behavioral characteristic that could be detected in *Drosophila* by a social space assay. Therefore, we used this assay to investigate the social interaction of *Trh* knockdown flies. The average distance to the closest neighbor showed a significant increase with *Trh* knockdown flies for both sexes (Fig. 1C, E). Cumulative frequency showed a more detailed distribution of every single fly in which a higher percentage of flies exhibited an increase in social distance (Fig. 1D, F). The same results were obtained in *Trh*^*01*^ flies, an indel mutant for *Trh* that failed to synthesize serotonin [30] (Fig. 1G), which scarcely had 5-HT in its bodies (Fig. 1H). *Trh*^*01*^ mutant flies displayed an increase in social spacing, as evidenced by the distance between an individual fly and its closest neighboring flies. Consistent with previous studies, at least a two-body length (~ 0.25 cm) was observed in most *Canton-S* wild-type flies in a social setting [26]. However, the *Trh*^*01*^ mutant flies showed a significantly far distance in a social group for both sexes (Fig. 1I–L), and a more obvious phenotype was detected in the female flies, as the *P* value (*P* = 0.0006, indicated by ^***^) showed a more significant difference (Fig. 1K). The graph of cumulative frequency suggested that 50% of the female flies settled close to 0.4 cm from their neighboring flies (Fig. 1L). Furthermore, the *Trh*^*01*^ mutant flies had a larger range of distances than the wild-type ones. The impact of locomotor defects can be excluded because no severe defective climbing activity was observed in *Trh* knockdown and mutant lines (Fig. 1M, N). These results indicate that a lack of serotonin severely affects the social interaction of *Drosophila*.

To further verify the effect of serotonin on the social interaction of *Drosophila*, we fed both *Trh*^*01*^ mutants and wild-type flies with 2 mg/mL 5-HTP for 3 days, which restored serotonin in the brains of *Trh*^*01*^ mutants, as observed by immunofluorescence (Fig. 1Q, R). We found that 5-HTP feeding rescued social avoidance in *Trh*^*01*^ mutants of either sex (Fig. 1O). Furthermore, a closer distance of individual flies was identified in *Canton-S* wild-type fed with 5-HTP as opposed to normal food feeding wild-type flies (Fig. 1P). Thus, serotonin plays an important role in the social interaction of *Drosophila*.

### Serotonin modulates the repetitive behavior of *Drosophila*

**Fig. 2:**
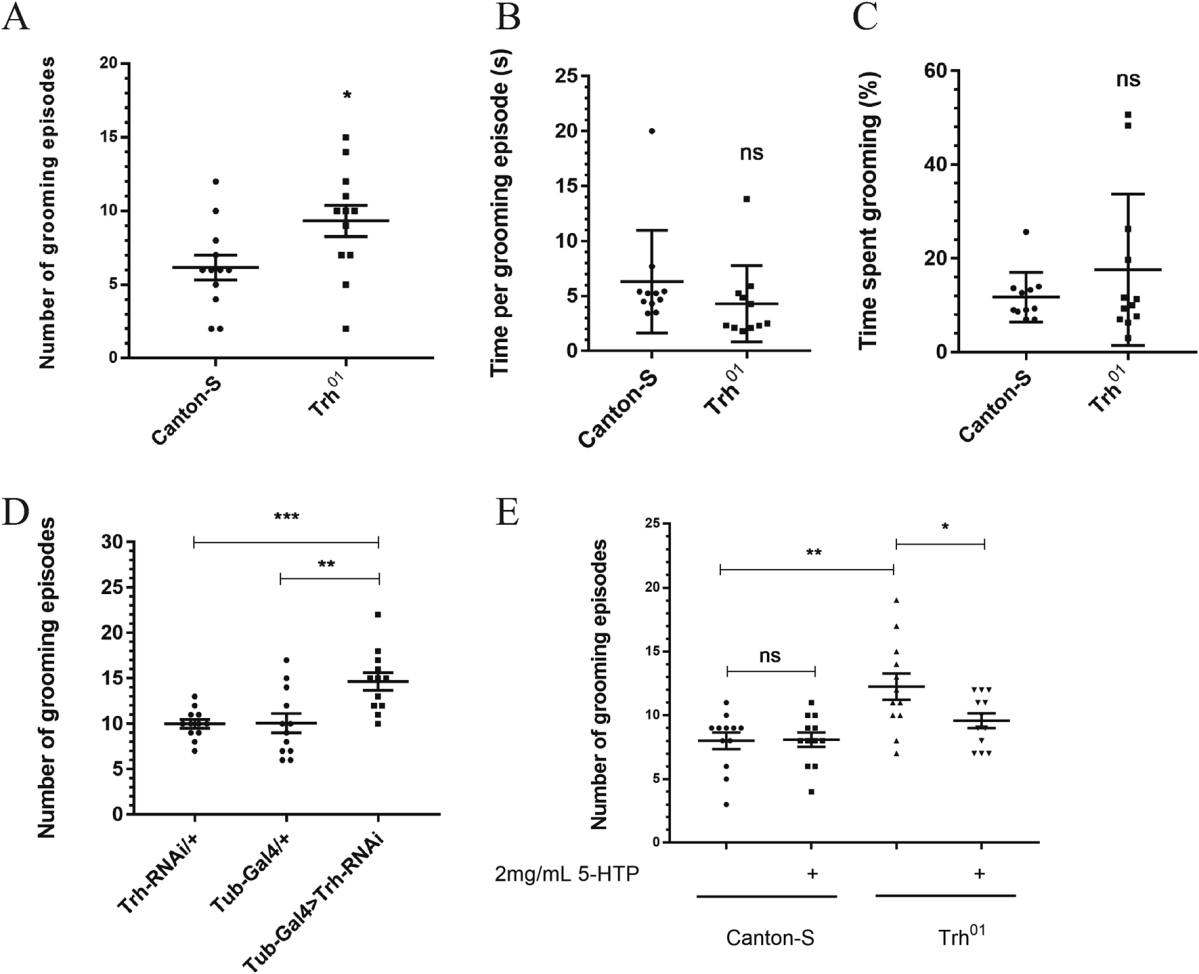
Serotonin regulates the repetitive behavior of *Drosophila*. *Trh*^*01*^ mutant male flies show more grooming numbers (**A**) but spent nearly the same time per grooming episode (**B**) as well as total time spent grooming (**C)** when compared with *Canton-S* control flies during a 5-min observation period (*n* = 10–15 flies for each genotype). **D** Grooming numbers of *Trh* knockdown male flies during a 5-min observation period. **E** After feeding 2 mg/mL 5-HTP for 3 days, *Trh*^*01*^ mutant male flies displayed decreased grooming numbers. Error bars are shown as the mean ± SEM. For all data, *p < 0.05, **p < 0.01, ***p < 0.001

Given that repetitive behavior is also a typical behavioral anomaly in ASD, we intended to determine whether serotonin affects the repetitive behavior of *Drosophila*. We detected the repetitive behavior of *Trh* knockdown and *Trh*^*01*^ mutant flies by calculating the number of grooming episodes in a 5-min period to ascertain whether serotonin plays a role in this process. The *Trh*^*01*^ mutant male flies displayed a markedly increased number of grooming episodes when compared with control flies (Fig. 2A), as did the female flies of the same genotype (Additional file 1: Fig. S1A). We also analyzed time spent per grooming episode and the total time devoted to grooming, but there was no significant differences (Fig. 2B, C). The same results were true of the data in the *Trh* knockdown files: the number of grooming episodes was significantly higher than that of both parental controls (Fig. 2D and Additional file 1: Fig. S1B). To examine whether this aggravated repetitive behavior caused by serotonin loss could be rescued by 5-HTP feeding, which would increase cerebral serotonin in mutant lines, we compared the number of grooming episodes between 5-HTP-fed *Trh*^*01*^ flies and their normal food-fed counterparts and found that the repetitive behavior of *Trh*^*01*^ flies was mitigated through 5-HTP intake (Fig. 2E and Additional file 1: Fig. S1C). In summary, serotonin modulates the repetitive behavior of *Drosophila*.

### Different serotonin receptors in regulating social behaviors

**Fig. 3:**
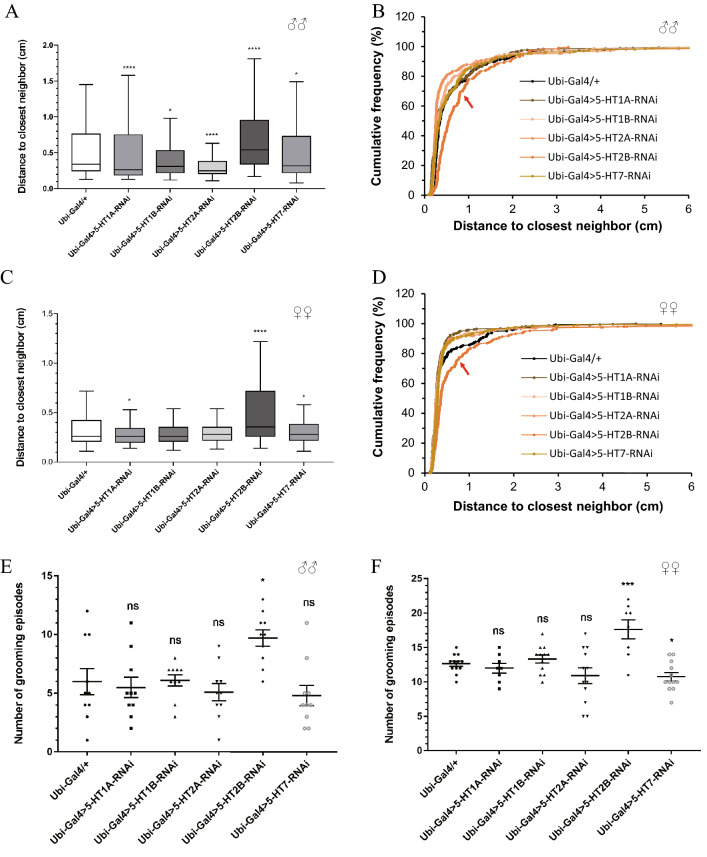
*5-HT2B* was required for normal social interaction and repetitive behavior in *Drosophila*. **A**, **C** Representative data show the distance to the closest neighbor of the five *Ubi-Gal4*-driven 5-HT receptor knockdown and *Gal4* control flies in males (**A**) or females (**C**). *p < 0.05, **p < 0.01, ***p < 0.001, ****p < 0.0001. **B**, **D** Cumulative probability distributions of males (**B**) and females (**D**) over 5-HT receptor knockdown and control flies. **E**, **F** Number of grooming episodes recorded by grooming assay of flies of all 5 receptors knocked down in both sexes (**E** for males, and **F** for females) in the 5-min observation period. Error bars are shown as the mean ± SEM (**E**) and (**F**)

Five receptors have been characterized to be 5-HT receptors in *Drosophila*: *5-HT1A*, *5-HT1B*, *5-HT2A*, *5-HT2B* and *5-HT7*, all of which belong to the superfamily of G-protein-coupled receptors [31]. The *5-HT1A*, *5-HT1B*, and *5-HT7* receptors couple to cAMP signaling cascades [32], while the *5-HT2A* and *5-HT2B* receptors lead to Ca^2+^ signaling in an inositol-1,4,5-trisphosphate-dependent manner [33]. To investigate which receptor participates in the regulation of social interaction and repetitive behavior in *Drosophila*, we knocked down all five 5-HT receptors by crossing *Ubi-Gal4* flies with *UAS-5-HT1A-RNAi*, *UAS-5-HT1B-RNAi*, *UAS-5-HT2A-RNAi*, *UAS-5-HT2B-RNAi*, and *UAS-5-HT7-RNAi* flies. The mRNA expression levels of these 5-HT receptors were significantly decreased in these flies (Additional file 2: Fig. S2A). No climbing defects were found in these receptor knockdown flies (Additional file 2: Fig. S2B). We analyzed the distance between an individual fly and its closest neighbors in these flies and found that only the *5-HT2B* knockdown flies had abnormal social space in both sexes with a significantly increased social distance compared with controls (Fig. 3A–D). Unsurprisingly, irregular repetitive behavior occurred in these knockdown flies, as shown by elevated grooming numbers in both male and female flies (Fig. 3E, D). Therefore, of the five receptors, *5-HT2B* appeared to be required for normal social behavior in *Drosophila*.

### Serotonin regulates social behaviors via the 5-HT2B receptor

**Fig. 4:**
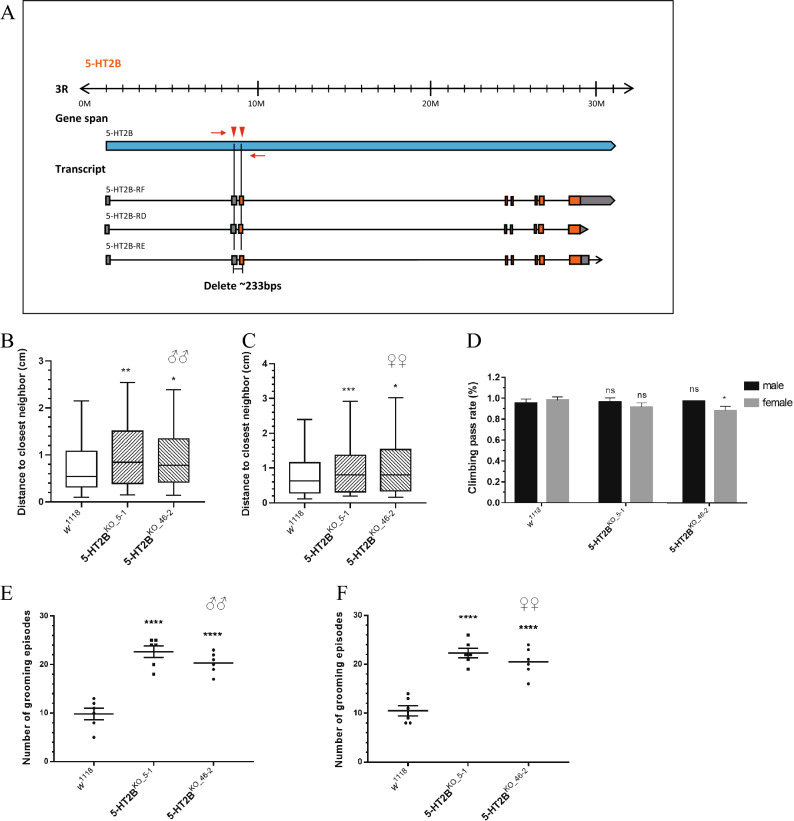
*5-HT2B* knockout flies showed further social distance and more severe repetitive behavior. **A** Schematic of two sgRNAs separately targeting the upstream region and first exon of the 5-HT2B locus. Orange boxes represent exons. Approximately 233 base pairs would be theoretically deleted when Cas9 is expressed in *Drosophila* germ cells. **B**, **C** Both 5-HT2B knockout lines showed further distance to their nearest neighbors in males (**B**) or females (**C**). *p < 0.05, **p < 0.01, ***p < 0.001, ****p < 0.0001. **D** No climbing defect was observed in 5-HT2B^KO_5−1^ male or female flies or 5-HT2B^KO_46−2^ males, while 5-HT2B^KO_46−2^ females showed a slight defect in climbing. **E**, **F** More grooming numbers were detected in these 2 5-HT2B knockout lines in both sexes. Error bars are shown as the mean ± SEM (**D**, **E** and **F**)

To further confirm the function of 5-HT2B in social behavior, we utilized the CRISPR/Cas9 approach to generate *5-HT2B* knockout lines. As shown in Fig. 4A, we designed 2 sgRNAs to target the *5-HT2B* gene: one was upstream of the first exon of this gene and the other in it. The 2 sgRNA plasmid mixtures were injected into the embryos carrying *nos-Cas9* transgene to obtain 223 bp deletion. After screening nine flies eclosed from injected embryos, we obtained two knockout lines with deletions of 224 bp and 247 bp (Additional file 3: Fig. S3A, B). For both knockout lines, male and female flies presented with a social abnormality phenotype, and the mutant flies stayed significantly farther than *w*^1118^ wild-type flies (Fig. 4B, C). Notably, the mutant lines also had remarkably elevated grooming numbers in the 5-min observation period (Fig. 4E, F). No severe climbing defects were found in these two mutants (Fig. 4D). Thus, possibly 5-HT2B is the major receptor that participates in serotonin-mediated social behaviors.

### Brain regions involved in regulating social interaction in  *Drosophila*

**Fig. 5:**
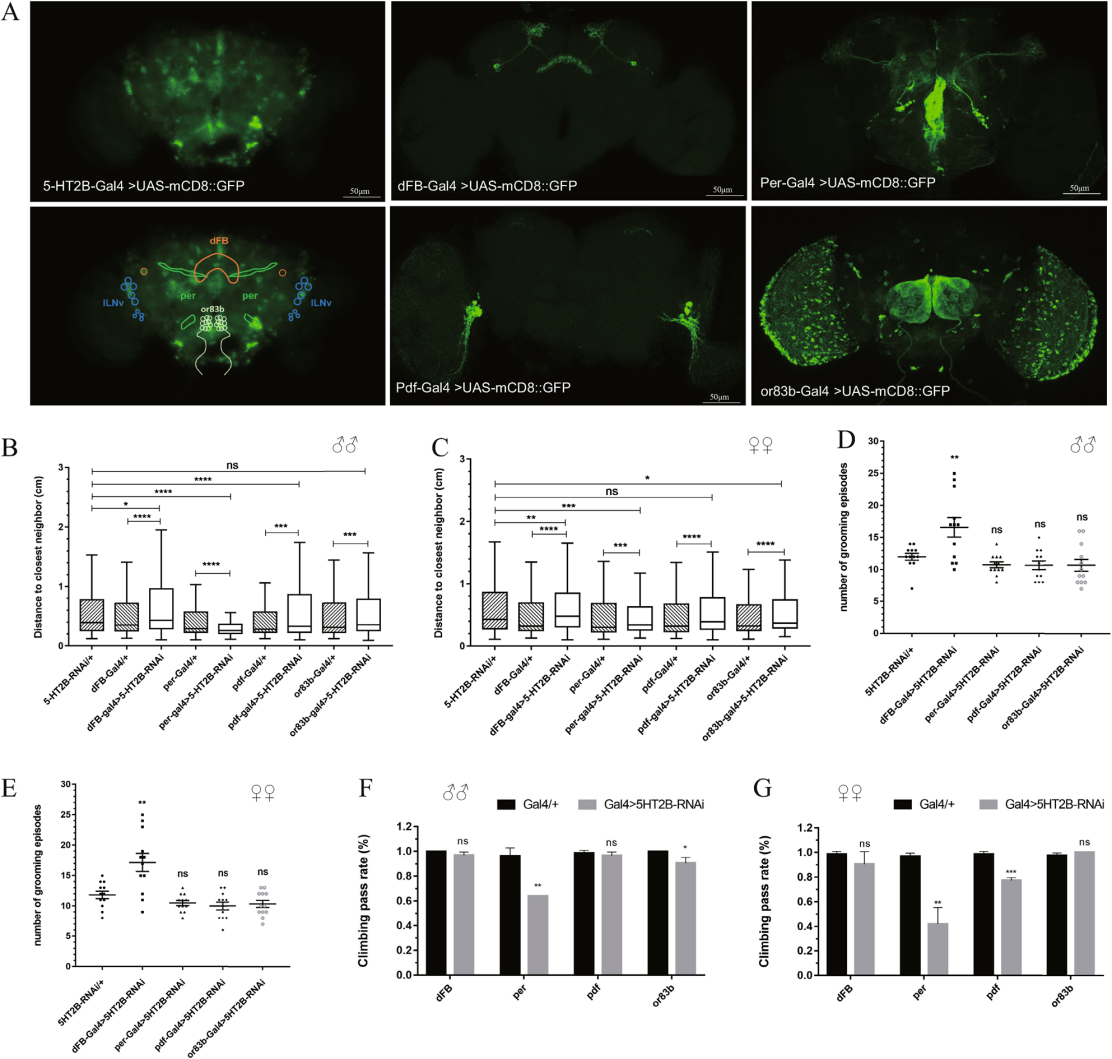
*5-HT2B* receptor in dFB neurons regulates the social interaction and repetitive behavior of *Drosophila*. **A** Expression pattern of *5-HT2B-Gal4*, *dFB-Gal4* (dorsal fan-shaped body neurons), *per-Gal4* (period neurons), *pdf-Gal4* (large ventral lateral clock neurons), *or 83b-Gal4* (odorant receptor neurons) in the brain is visualized by *UAS-mCD8: GFP* (green). Areas located by different neurons are labeled: dFB (orange), per (dark green), ILNvs (blue), or83b (light green). **B**, **C** Quantification of the distance to the closest neighbor of targeted knockdown of *5-HT2B* in different parts of brain neurons by expression of *UAS-5-HT2B-RNAi* under control of different Gal4. The distances between each fly of dorsal fan-shaped body-specific *5-HT2B* knockdown (*dFB-Gal4* > *5-HT2B-RNAi*) flies were significantly longer than those of control flies. *p < 0.05, **p < 0.01, ***p < 0.001, ****p < 0.0001. **D**, **E** Number of grooming episodes of different Gal4-specific 5-HT2B knockdown flies. Both males and females of the *dFB-Gal4* > *5-HT2B-RNAi* genotype showed more grooming numbers. **F**, **G** Climbing ability of *5-HT2B* knockdown flies detected by negative geotaxis assay. Error bars are shown as the mean ± SEM (**D**-**G)**

In *Drosophila*, more than 500 *5-HT2B*-positive neurons are distributed in the brain, whose axons and dendrites are located in the central complex, the olfactory lobe, the optic lobe, the sub-esophageal ganglion and the ventrolateral protocerebrum (Fig. 5A). The same expression pattern of *5-HT2B* has been identified by Qian and colleagues [31]. Given our observation that *5-HT2B* receptor defects could affect social interaction, we asked which brain regions or subsets of neurons specifically participate in normal social interaction in the *Drosophila* brain.

Since a small subset of *5-HT2B*-expressing neurons in the dorsal fan-shaped body (dFB) regulate sleep homeostasis in *Drosophila *[31], we asked whether these serotoninergic neurons could also regulate the social interaction behaviors of flies. The social space assay was utilized to assess the social behaviors of *dFB-Gal4* > *UAS-5-HT2B-RNAi* flies in which *5-HT2B* was specifically knocked down in dFB and a strikingly farther distance was recognized between these genotypic flies (Fig. 5B, C). Intriguingly, the grooming number in the 5-min period was larger in *dFB-Gal4* > *UAS-5-HT2B-RNAi* flies than in both parental controls (Fig. 5D, E). Considering the role of *5-HT2B* in the circadian rhythm, we utilized *per-Gal4* flies to specifically knockdown *5-HT2B* in the clock gene period. Surprisingly, these knockdown flies exhibited a closer distance between each other (Fig. 5B, C). As *per-Gal4* > *UAS-5-HT2B-RNAi* flies had significant locomotion defects (Fig. 5F, G), more time was given for the flies to settle down while receiving social space assay. A previous study showed that large ventral lateral clock neurons (lLNvs) are vital for maintaining the normal circadian rhythm associated with arousal and sleep [34]. As the *5-HT2B* receptor was also expressed in lLNvs (Fig. 5A), *pdf-Gal4* was used to specifically knockdown *5-HT2B* in lLNvs. As shown in the chart, the distance of the fly to its nearest neighboring fly increased when compared with its *Gal4* parental controls but decreased for its RNAi parental controls (Fig. 5B, C). Moreover, no repetitive behavior defects were detected in *pdf-Gal4* > *UAS-5-HT2B-RNAi* flies (Fig. 5D, E). Therefore, we excluded these neurons as candidates that may regulate the social behaviors of flies. Synaptic connections between serotonin synthesizing neurons and olfactory receptor neurons have been established in the fruit fly brain and contribute to behaviors related to olfaction, such as learning and memory [35]. In addition, olfactory sensory neurons were shown to mediate social avoidance in *Drosophila *[36], and the *5-HT2B* receptor was distributed in olfactory neurons (Fig. 5A). To examine whether the *5-HT2B* receptor functions in the olfactory neurons to regulate the social interaction of flies, we knocked down *5-HT2B* in olfactory neurons by *Or83b-Gal4*. Similar to *pdf-Gal4* knockdown flies, *r83b-Gal4* > *UAS-5-HT2B-RNAi* flies showed normal grooming numbers and intermediate nearest-neighboring distances when compared with parental controls (Fig. 5B–E), indicating that *5-HT2B* receptors in olfactory neurons were not responsible for the social behaviors of *Drosophila*.

In summary, serotonin regulates the social interactions and repetitive behaviors of *Drosophila* through *5-HT2B* receptors in dFB neurons, and the functions of *5-HT2B* receptors in the social interaction of *Drosophila* are independent of circadian rhythm or olfactory neurons.

## Discussion

Increasing incidence of autism has brought great pressure to the family and society. The pathogenesis of autism and related drug targets are of great significance for the treatment of autism. A strong relationship between serotonin and autism has been reported in the literature. In 1961, elevated blood serotonin levels were found in infantile autism [37], and a further study reported that more than 25% of autistic individuals had higher serotonin blood levels [6]. Therefore, serotonin levels have been a biomarker in autism researches. Determining the association between serotonin and autism is fundamental to the treatment of autism. Reportedly, the 5-HT contained in whole blood is almost completely contained in platelets [38]. However, the vast majority of 5-HT produced in the periphery is unable to cross the mature blood brain barrier and interact with neural tissue [39]. Instead, 5-HT found in the brain is produced by serotonergic neurons in the midbrain and hindbrain [40]. Despite the high blood serotonin ( or hyperserotonemia), studies also show low levels of serotonin in the brains of autistic children, as decreased uptake of tryptophan, known as the precursor of 5-HT, and reduced 5-HT synthesis were observed in the brains of autistic children [7–9]. Moreover, McDougle and colleagues reported that decreased synaptic 5-HT caused by tryptophan depletion worsened repetitive behaviors and irritability in autism [41]. The same phenomenon was also observed in mice lacking *TPH2*, which is responsible for central 5-HT synthesis. These mice had decreased ultrasonic vocalizations and sniffing of social odors, as well as defective social memory and inflexible cognition [42–44]. Other studies have shown that maternal virus exposure or immune activation results in decreased brain 5-HT levels or abnormal 5-HT neurons [10, 11]. To investigate the association between serotonin and autism, we analyzed the social interaction of flies by a social space assay and individual repetitive behavior by a grooming assay and found that *Trh*^*01*^ flies showed significantly increased social distance and grooming numbers when normally fed. Such 2 abnormal behaviors were rescued by feeding *Trh*^*01*^ flies with 5-HTP, suggesting a vital role of serotonin in social behavior regulation. We noticed that in wild-type *Canton-S* there was no change in serotonin contents by feeding of serotonin, while social behavior was affected by feeding of serotonin in *Canton-S*. It is postulated that some other mechanisms may underline regulating the social behavior of 5-HTP fed wild-type flies.

A postmortem study showed decreased 5-HT2A and 5-HT1A binding in ASD [45]. Another 5-HT receptor, 5-HT1B, showed its necessity in postsynapse to establish social preference [46]. However, Veenstra-VanderWeele et al. reported that the expression of the Ala56 variant of the 5-HT transporter gene *SLC6A4* in mice, whose association with compulsive behaviors has been detected, leads to hyperserotonemia, more brain 5HT clearance, and higher 5-HT2A receptor sensitivity [47]. They also identified 5-HT1B receptor binding in these mice and a paralleling phenomenon in which 5-HT1B receptor binding was increased in the orbitofrontal cortex [48]. Regarding the 5-HT7 receptor, although one study demonstrated an absence of correlation between the *5-HT7* gene polymorphism and ASD [49], this type of receptor has been shown to modulate behavioral flexibility [50], exploratory behavior [51], mood disorders [52] and epilepsy [53], including core and comorbid symptoms of ASD. These findings confirmed the involvement of 5-HT7 receptors in ASD [54]. Nevertheless, it seems that future work is warranted to investigate the roles of each 5-HT receptor in ASD. Meanwhile, which 5-HT receptors dominate the regulation of social and stereotyped behavior in autistic children is still shrouded in mystery. In this study, when we knocked down all 5 serotonin receptors in *Drosophila*, the *5-HT2B* knockdown flies displayed a significant rise in social space, as well as increased grooming numbers for both sexes, proving that 5-HT2B has a significant function in regulating social and repetitive behaviors. The role of *5-HT2B* in autism-like behaviors was validated by *5-HT2B* knockout flies, which were generated through the CRISPR/Cas9 system. Moreover, with the help of different *Gal4* lines, we discovered that the *5-HT2B* receptor in dFB neurons was important for normal social interaction in *Drosophila*. Additionally, these neurons also affected the repetitive behaviors of flies, since knockdown of 5-HT2B in dFB neurons resulted in elevated grooming numbers in a 5-min period of time.

In the human body, there are two distinct sources of 5-HT. In total, 95% of 5-HT within us is generated by the gut [55], where most serotonin is produced by TPH1 in enterochromaffin (EC) cells and a small portion is produced by TPH2 in myenteric serotonergic neurons of the gastrointestinal (GI) tract. As for neuronal 5-HT, nearly all neuronal 5-HT production is dependent on TPH2 in serotonergic neurons. In *Drosophila*, only one TRH exists. In addition to being highly expressed in the adult brain, TRH is also expressed in the gut, especially in enterocytes (ECs), according to Drosophila Gut Data Sets from the website Single-Cell RNA-seq (URL: https://www.flyrnai.org/scRNA/gut/). In this study, we only focused on neuronal functions of TRH without involving GI TRH, which may result in a relatively incomprehensive observation. GI 5-HT regulates a variety of intestinal functions [55] and functions as a link between the gut–brain–microbiome axis in ASD [56]. Several enteric mucosa-associated Clostridial species have been assumed to be associated with levels of serotonin [57]. Therefore, future studies could be performed to evaluate whether *Trh* functions in the gut and microbes that inhabit the intestine. Furthermore, the function of microbes in ASD development should also be investigated.

**Fig. 6:**
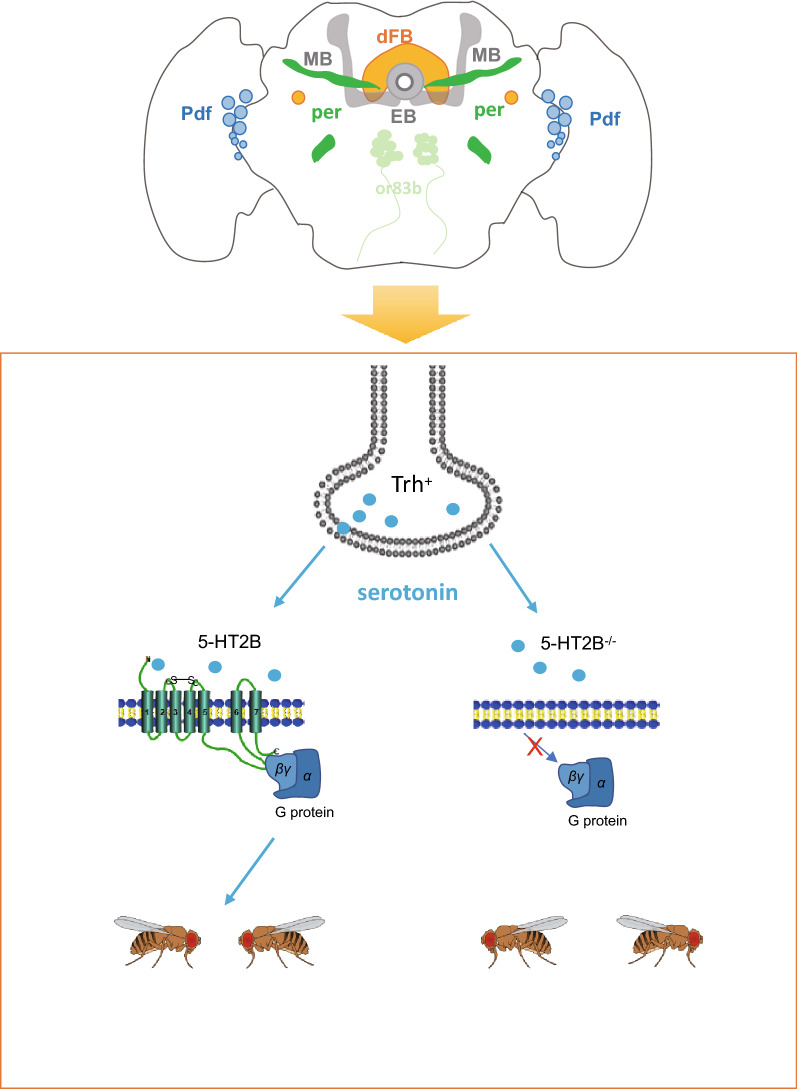
Schematic model for serotonin and its receptor 5-HT2B on autism-like behaviors. The *Drosophila* brain with main areas is in frontal view. Each color represents a distinct type of neuron: dFB (orange); per (dark green); pdf (blue); or83b (light green); MB, mushroom body and EB, ellipsoid body (dark gray). Normally, serotonin synthesized and released from presynaptic neurons located in the dorsal fan-shaped body binds to 5-HT2B receptors and activates G proteins postsynaptically, which maintains normal social behavior in *Drosophila*. When reduced in dFB neurons, 5-HT2B receptors could not activate downstream G proteins, leading to stereotyped behavior and abnormal social interaction in flies

In brief, our findings reveal a close relationship of serotonin levels and the 5-HT2B receptor to the social behaviors of *Drosophila,* including social interaction and repetitive behavior. With lower levels of serotonin, *Drosophila* showed autism-like behaviors, involving further distance to their neighbors and increased grooming numbers. In addition, 5-HT2B receptors in dFB neurons dominated the function of serotonin in regulating the two autism-like behaviors (Fig. 6). Our study further established *Drosophila* as a model for the detection of diseases with strong social behavior abnormalities. We conclude that 5-HT2B promises to be a possible therapeutic target for the treatment of ASD.

## Supplementary Information

**Additional file 1: Fig. S1.** Repetitive behavior is regulated by serotonin in females. Grooming numbers of *Trh*^01^ mutant (**A**) or *Trh* knockdown (**B**) female flies during a 5-min observation period. (**C**) Feeding 2 mg/mL 5-HTP for 3 days rescued excessive grooming behavior of *Trh*^*01*^ mutant male flies during a 5-min observation period (*n*= 10-15 flies for each genotype). Error bars are shown as the mean ± SEM. For all data, *p<0.05, **p<0.01, ***p<0.001, ****p<0.0001.

**Additional file 2: Fig. S2.** Ubiquitous knockdown of serotonin receptors has no effect on the climbing ability of flies. (**A**) qRT-PCR analysis of the mRNA expression of all five serotonin receptor knockdown lines. (**B**) Climbing ability of serotonin receptor knockdown flies detected by a negative geotaxis assay. Error bars are shown as the mean ± SEM. For all data, *p<0.05, **p<0.01, ***p<0.001.

**Additional file 3: Fig. S3.** Verification of *5-HT2B* knockout flies. (**A**) Molecular verification of *5-HT2B* knockout flies by PCR analysis. Only one band was seen in *w*^*1118*^ controls, but a lower band could exist when a fragment of approximately 233 bp was deleted from the *5-HT2B *locus. (**B**) The PCR-Sanger sequencing results of two reserved homozygous *5-HT2B* knockout lines. The results show that the *5-HT2B*^*KO_5-1*^ line has a 224 bp deletion in the first exon of the 5-HT2B genome, and the 5-HT2B^KO_46-2^ line has a larger deletion as well as a 2 bp insertion.

**Additional file 4: Table. S1** Information on all primers used in this study.

## Data Availability

Not applicable.

## Abbreviations

5-HT: 5-Hydroxytryptamine
ASD: Autism spectrum disorder
WB: Whole blood
PRP: Platelet-rich plasma
lLNvs: Large ventral lateral clock neurons
dFB: Dorsal fan-shaped body
5-HTP: 5-Hydroxytryptophan
EC: Enterochromaffin
GI: Gastrointestinal
ECs: Enterocytes
